# False memory for idiomatic expressions in younger and older adults: evidence for indirect activation of figurative meanings

**DOI:** 10.3389/fpsyg.2014.00764

**Published:** 2014-07-21

**Authors:** Jennifer H. Coane, Claudia Sánchez-Gutiérrez, Chelsea M. Stillman, Jennifer A. Corriveau

**Affiliations:** ^1^Memory and Language Lab, Department of Psychology, Colby CollegeWaterville, ME, USA; ^2^Department of Languages, Linguistics, and Translation, Université LavalQuebec, QC, Canada; ^3^Department of Psychology, Georgetown UniversityWashington, DC, USA; ^4^Department of Psychology, University of ConnecticutStorrs, CT, USA

**Keywords:** idioms, figurative language, memory, false memory, aging

## Abstract

Idiomatic expressions can be interpreted literally or figuratively. These two meanings are often processed in parallel or very rapidly, as evidenced by online measures of idiomatic processing. Because in many cases the figurative meaning cannot be derived from the component lexical elements and because of the speed with which this meaning is accessed, it is assumed such meanings are stored in semantic memory. In the present study, we examined how literal equivalents and intact idiomatic expressions are stored in memory and whether episodic memory traces interact or interfere with semantic-level representations and vice versa. To examine age-invariance, younger and older adults studied lists of idioms and literal equivalents. On a recognition test, some studied items were presented in the alternative form (e.g., if the idiom was studied, its literal equivalent was tested). False alarms to these critical items suggested that studying literal equivalents activates the idiom from which they are derived, presumably due to spreading activation in lexical/semantic networks, and results in high rates of errors. Importantly, however, the converse (false alarms to literal equivalents after studying the idiom) were significantly lower, suggesting an advantage in storage for idioms. The results are consistent with idiom processing models that suggest obligatory access to figurative meanings and that this access can also occur indirectly, through literal equivalents.

## False memory for idiomatic expressions and their literal equivalents in younger and older adults

Idioms, such as *kick the bucket*, are prevalent in everyday speech and are examples of figurative language. In addition to the figurative meaning, many idioms also have a literal interpretation—in the example above, to physically kick a pail or bucket. Whereas the latter is given by the meaning of the component words, the figurative meaning of an idiom cannot always be inferred by the sum of its component words, but requires prior knowledge that this particular combination of lexical units conveys a different meaning. Therefore, in order to efficiently access the idiom's figurative meaning, this knowledge must have been previously learned and stored in memory.

### Literal and figurative meanings in idiom processing

A central issue in research on idiomatic expressions is to explain how both literal and figurative meanings are processed. One way to examine this is to measure the extent to which literal word meanings are activated during processing. Across different models, the presumed degree of activation of literal word meanings varies substantially. For example, the Lexical Representation Hypothesis (Swinney and Cutler, 1979) assumes parallel processing of literal and figurative analyses, whereas the Idiom List Hypothesis (Bobrow and Bell, 1973) assumes no literal activation occurs, because idioms are stored separately in the lexicon as large lexical units. Recently, Rommers et al. (2013) reported behavioral and electro-physiological data consistent with unitary representations (cf. Bobrow and Bell, 1973). Specifically, in their study, Rommers et al. found no evidence of semantic activation of the literal word meanings when the idioms were presented in biasing contexts, suggesting that top-down processes, such as those given by context and expectancies, might be sufficient to “turn off” word-level semantic processing.

However, it is important to note that other studies, which present idioms in isolation, find evidence of literal word-level processing. As such, some models, like that proposed by Cutting and Bock (1997), suggest that idioms are stored as units, but that activation can spread from the idiom to related word meanings of the composing lexical units that compose the given idiom. Other hybrid models include the Direct Access Hypothesis (Gibbs, 1980), according to which literal analysis only occurs when figurative analysis fails. Finally, the Configuration Hypothesis (Cacciari and Tabossi, 1988) suggests that literal analysis is performed on incoming lexical items until the “idiomatic key” (i.e., the point in which the idiom is recognized as such) is encountered, after which time, only figurative processing occurs. In sum, there is still a lack of consensus concerning how idioms are processed.

In part as a test of these conflicting hypotheses, a substantial body of literature (e.g., Gibbs et al., 1989; Cacciari and Glucksberg, 1991; Titone and Connine, 1994a, 1999; Libben and Titone, 2008) has investigated when and how literal and figurative meanings are accessed and retrieved. This research has predominantly relied on speeded tasks, such as lexical decision (Cacciari and Tabossi, 1988), or production tasks (Cutting and Bock, 1997; Sprenger et al., 2006). In general, there is evidence that the figurative meaning is available immediately or soon after presentation of the idiom, depending on how strongly the current context biases interpretation (Cacciari and Tabossi, 1988). Additional studies have examined how speakers understand idioms and the relations between the individual words and the figurative meaning (e.g., Gibbs et al., 1989; Cacciari and Glucksberg, 1991; Libben and Titone, 2008). Several studies suggest that the idiomatic interpretation precedes the literal interpretation (Ortony et al., 1978; Gibbs, 1980; Cacciari and Tabossi, 1988). However, although both literal and figurative meanings are processed, it is difficult to tease these representations apart, in part because of the speed with which such processing occurs (for example, Cacciari and Tabossi, 1988, suggested that figurative meanings are available approximately 300 ms after presentation for non-transparent idioms and immediately for predictable idioms). The evidence reviewed above suggests that, although it is still not clear exactly when and under what conditions figurative and literal meanings are accessed, the figurative meaning does need to be stored in semantic memory to support rapid and efficient processing of written and spoken language.

Accessing stored meaning is likely more important for those idioms in which the figurative meaning cannot be derived from the component words (e.g., *kick the bucket*). These items are said to lack in transparency compared to other idioms, such as *bite your tongue*, in which the figurative meaning of “not speaking” can be derived from the component words. However, even for the less transparent idioms, accessing a stored meaning for the complete idiomatic phrase would presumably be less effortful and more efficient than computing the meaning from the component words.

### Interactions between semantic and episodic memory

Knowledge about the meaning of words is stored in semantic memory (e.g., Balota and Coane, 2008); knowledge about idioms is presumably also stored in this system. Semantic memory is generally defined as the storehouse of knowledge that includes, among other things, factual and conceptual knowledge and the mental lexicon. Retrieval from semantic memory is often assessed using visual word recognition tasks, such as lexical decision, in which participants indicate whether a letter string is a word (i.e., an entry in the lexicon) or not. Lexical access occurs when a lexical entry is identified—i.e., the moment when the item's meaning becomes available (Balota, 1990). Thus, lexical tasks offer insights into how information is stored in and retrieved from semantic memory, as well as how it is organized.

The effects of semantic and associative relatedness are well-established in word recognition (see McNamara, 2005, for a review) and memory literatures. A large body of research examining semantic and associative priming suggests that processing words stored in the same semantic networks results in changes in the accessibility or activation of related word nodes, resulting in faster response latencies and greater accuracy in lexical tasks such as lexical decision (i.e., word-nonword decisions; e.g., Meyer and Schvaneveldt, 1971; Neely, 1977) and pronunciation. Thus, processing an item such as *pail* will result in the indirect activation of related items such as *water* or *bucket* (e.g., Collins and Loftus, 1975). Importantly, this activation is assumed to occur automatically outside of intentional or volitional processes (see Neely, 1977).

In contrast to semantic memory, episodic memory refers to the type of memory involved in the retention and retrieval of specific events or episodes (Tulving, 1972; Szpunar and McDermott, 2008). Typically, episodic memory is tested by assessing participants' ability to correctly identify items studied in a specific context or list, often requiring discrimination from similar, but non-studied, foils. Although often conceptualized as separate systems (e.g., Tulving, 1972), there is evidence that these two stores interact with one another influencing performance in a variety of ways (see Balota and Coane, 2008 for a review).

Perhaps the most well-known evidence for semantic and episodic memories interfering/interacting with one another comes from research on false memory, more specifically from studies using the Deese–Roediger–McDermott (DRM; Deese, 1959; Roediger and McDermott, 1995) paradigm. In this paradigm, participants study lists of related items (e.g., *bed*, *rest*, *tired*, *dream*) that converge upon a non-presented related critical item (CI; e.g., *sleep*). On memory tests, participants falsely recall or recognize the CI. These false memories are robust and persist in the face of warnings or instructions to adopt conservative response strategies (Gallo, 2010). According to the activation-monitoring account (Roediger et al., 2001a), false memories occur because converging activation in semantic networks from the list items to the CI increases the activation or accessibility of the CI. At retrieval, participants misattribute the source of this increased accessibility to the episodic study event. Importantly, this activation occurs automatically when as few as three or six list items are processed (Marsh et al., 2004; Coane and McBride, 2006; Coane et al., 2007) and the effects are quite persistent in direct memory tests (Meade et al., 2007).

Additional evidence for the influence of schematic or semantic processing in episodic memory tests comes from studies such as Jenkins (1974; see also Bransford and Franks, 1971). Here, participants studied sentences such as “the girl broke the window” and “the girl lives next door.” On a memory test, participants erroneously endorsed sentences like “the girl who lives next door broke the window,” suggesting participants were relying on the gist or integrative meaning of the studied sentences. Furthermore, as demonstrated by Sachs (1967), verbatim memory for prose passages declines rapidly, whereas the meaning or gist persists. Thus, while associative semantic memory networks allow for efficient processing of linguistic material, this efficiency can come at the cost of accurate/precise *episodic* memory. In other words, activation of similar or related information in semantic memory can result in errors in episodic tasks. As an extension, these errors can be used to draw inferences about the underlying nature of semantic networks that give rise to these errors.

### Processing of idioms in semantic and episodic memory

Within the context of idiom processing and comprehension, processes in semantic memory presumably support the stored figurative interpretation, whereas episodic memory traces might support prior experiences with a particular idiom. Indeed, the speed with which figurative meanings are derived in idiom processing is consistent with rapid and automatic retrieval of meaning from semantic memory stores (e.g., Neely, 1977; Balota and Coane, 2008).

In the present study, we examined how episodic and semantic memory systems interact in the processing of idiomatic expressions. The first assumption we made was that figurative meanings are stored in semantic memory and activated or accessed when an idiom is processed. We further assumed that the literal meanings of the component words are stored in associative networks in semantic memory (e.g., Anderson and Bower, 1973; Collins and Loftus, 1975). For example, *bucket* and *pail* are semantically related and are, therefore, part of the same associative network.

Here we developed a memory task similar to the ones described above to examine idiom processing. If literal and figurative meanings of idioms are activated in a somewhat obligatory fashion (e.g., Westbury and Titone, 2011), then both meanings should be stored as part of a complex memory trace and undergo temporary changes in accessibility or activation. Thus, lexical access of one item, such as *bucket* should activate its neighbors in semantic memory, such as *pail* and vice versa. Borrowing from the false memory literature, we used false alarms (incorrect “old” responses to non-studied items) to measure activation or accessibility. Participants studied lists of idioms and literal equivalents (LE; i.e., non-idiomatic phrases that preserve the literal meaning of or are synonymous with an idiom, such as *kick the pail* instead of *kick the bucket*). On a subsequent memory test, the critical foils were the idiomatic version of studied literal equivalents (e.g., *kick the pail* in the study list and *kick the bucket* in the test list) and literal equivalents of studied idioms (e.g., *hold your horses* in the study list and *hold your ponies* in the test list). The main question was whether studying a literal equivalent like *kick the pail* increased the accessibility of *kick the bucket* and vice versa because of the relationship between *pail* and *bucket*. If the literal meaning of an idiom is the sum of the words comprising the idiom, principles of spreading activation predict that phrases or words with similar meanings would also be activated and more accessible in semantic memory (e.g., Jenkins, 1974; Collins and Loftus, 1975; Neely, 1977). Thus, if individual words are activating their semantic associates, *bucket* and *pail* should activate one another. Importantly, the indirect activation of *kick the bucket* should result in the formation of a complex memory trace that includes the non-presented figurative sense of the phrase. The critical comparison was between false alarm rates to idioms when the corresponding LE was studied and false alarm rates to LEs when the corresponding idiom was studied.

Equivalent error rates in these two conditions would suggest that participants encoded and stored the literal meaning or gist, with little or no influence of the stored figurative meaning. In other words, to the extent that *bucket* and *pail* are related, the two phrases should be stored similarly in episodic memory and should result in comparable false alarm rates. However, if studying *kick the pail* activates *kick the bucket* during encoding through the shared meaning of the noun phrases, then the figurative meaning in semantic memory might also be stored, consistent with notions that figurative processing is obligatory (e.g., Westbury and Titone, 2011). Thus, errors to *kick the bucket* when *kick the pail* is studied should be higher than errors to *kick the pail* when *kick the bucket* is studied, because of converging episodic and semantic traces.

In addition, if the figurative meaning is accessed automatically during encoding, one might expect fewer errors to *kick the pail* after studying *kick the bucket*, because the figurative meaning would be stored, perhaps more strongly than the literal meaning or verbatim surface information because of the pre-existing trace in semantic memory. This would suggest that the idiom was stored holistically (like a large lexical unit; Swinney and Cutler, 1979) or as a figurative gist trace (Sachs, 1967). As such, it might not activate a semantically related phrase that does not share the figurative meaning. This would be consistent with non-compositional models (e.g., Bobrow and Bell, 1973; Swinney and Cutler, 1979; Gibbs, 1980), in that the idiomatic expression is treated much like a unique lexical entry and the component words of idiomatic expressions are not processed fully as lexical units. Such a finding would also be consistent with prior work showing an advantage for figurative over literal processing (e.g., Ortony et al., 1978; Gibbs, 1980; Rommers et al., 2013).

Furthermore, as reported by Popiel and McRae (1988), idioms as a whole vary in the rated familiarity of their literal and figurative meanings. For example, *break the ice* is fairly commonly used, and thus familiar, in both senses, whereas *pick someone's brains* was rated as high in figurative meaning and low in literal meaning. Interestingly, even for the idioms rated highly on both usages, figurative meanings were generally rated as more familiar than literal meanings, suggesting that figurative meanings might be highly accessible, even when LEs are encoded. Thus, it is likely, for the familiar idioms used in the present study, that the figurative meanings are relatively “easy” to access or activate and the increased activation would likely influence performance on the memory task.

In addition to examining whether literal and figurative meanings provide bi-directional access to one another, we examined whether the effects were age invariant. Language experience is known to vary with age (e.g., Burke and Shafto, 2008). Compared to college-aged adults, older adults (i.e., individuals over 60) have an average of 45–50 years of additional experience in their native language, which might translate into differential processing of idiomatic expressions. To date, however, relatively little work has examined idiom processing in older populations (but see Westbury and Titone, 2011). Older adults generally show preservation or improvement in semantic memory tasks (see Balota et al., 2000) compared to younger adults, although there is also evidence that aging results in difficulties in lexical retrieval (Burke et al., 1991; Abrams et al., 2007). Older adults also tend to have difficulties inhibiting competing or irrelevant information (e.g., Hasher and Zacks, 1988) and often rely heavily on familiarity at the expense of item-specific recollection (e.g., Tun et al., 1998; Dennis et al., 2007). Therefore, examining this age group's performance could offer valuable insight into how idioms are remembered, how episodic and semantic traces might affect memory performance, and how age and consequent increases in experience with a language may affect these processes.

## Method

### Participants

Twenty-five college-aged students (*M*_age_ = 19.4, *SD* = 1.2, range 18–21; *M*_education_ = 13.6, *SD* = 1.49) from a liberal arts college in the Northeastern United States participated in exchange for $5 or course credit. Thirty-two healthy older adults were recruited from the surrounding community and were compensated at a rate of $5/h. Older adults had a mean age of 68.3 years (*SD* = 5.33) and an average education of 16.3 years (*SD* = 3.00). Older adults also completed a battery of cognitive tests, including measures of working memory (Operation Span task; Unsworth et al., 2005), vocabulary (Shipley, 1940), executive control (Trails B; Reitan, 1958), and processing speed (Digit Symbol Substitution Test, DSST; Wechsler, 1997). Scores on the cognitive battery are presented in Table 1. The Institutional Review Board at Colby College approved the study.

**Table 1:** **Cognitive battery scores for older adults**.

**Measure**	**Mean**	***SD***	**Range**
Operation span	18.00	8.34	4–34
Shipley vocabulary	34.64	4.49	20–40
DSST	50.47	8.59	28–76
Trails B	102.59	52.44	34–291

### Materials

Eighty commonly used idiomatic expressions of the form “Verb + Noun” phrase were selected (e.g., *kick the bucket, hold your horses*). In a pilot study, 20 undergraduate students (who did not participate in the experiment) provided familiarity ratings on a scale of 1 (not at all familiar) to 5 (extremely familiar) to all 80 idioms. The 60 most familiar idioms were selected for the experiment. For each idiomatic expression (hereafter *intact idiom*, denoted II), a modified literal phrase (hereafter *literal equivalent*, denoted LE) was created by substituting the noun in the II for another sharing the same literal meaning (e.g., *kick the pail, hold your ponies*). This resulted in 60 LEs, which retained the same literal meaning as the IIs, but no longer had a figurative meaning (see Appendix for a full list of stimuli).

The study list consisted of 20 IIs and 20 LEs. The test list included 60 items, half of which were presented in the same format (10 II-II and 10 LE-LE). The correct response to these items was “old.” The other 20 items from the study list constituted the critical foils and the alternate form was presented at test relative to the study phase (10 II-LE and 10 LE-II). In addition, 20 (10 II and 10 LE) fillers that had not been presented in either form were included to provide baseline false alarm rates. The correct response to the latter two categories was “new.” Items were counterbalanced across conditions. See Table 2 for examples.

**Table 2:** **Sample stimuli used in the encoding and retrieval phases of the experiment**.

**Study**	**Test**	**Condition**	**Correct response**
Kick the bucket	Kick the bucket	II-II	Old (hit)
Bite the dust	Bite the dirt	II-LE	New (critical false alarm)
Hold your ponies	Hold your horses	LE-II	New (critical false alarm)
Bury the ax	Bury the ax	LE-LE	Old (hit)
–	Smell a rat	Foil II	New (filler false alarm)
–	Spill the peas	Foil LE	New (filler false alarm)

*II, intact idiom; LE, literal equivalent*.

### Procedure

Participants were tested individually or in small groups of up to 4 individuals. Each participant was seated at an individual computer station. The experiment was administered via E-Prime software (Schneider et al., 2002). Participants were told they would be studying a list of phrases for an unspecified memory test. The 40 items in the study list were presented one at a time at a rate of 4 sec/item with a 500 ms inter-stimulus interval. The order of items was randomized anew for each participant. A 5 min unrelated filler task (i.e., Sudoku puzzles) was administered between the encoding phase and the recognition test. The instructions for the test emphasized that a phrase should be endorsed as studied only if it had appeared in the exact same form. As an example, participants were told that if they had studied *pet the dog* and the test included *caress the dog*, the correct answer was “new.” The 60 phrases appeared one at a time in random order. The test phase was self-paced. After the recognition test, all 60 idioms were presented (in the intact form) for a familiarity rating, using the same scale as the pilot study. The entire experiment lasted less than 30 min.

## Results

The data from eight older and one younger adult were omitted from analyses because of computer errors or failure to follow instructions (e.g., pressing the wrong keys). The analyses thus include data from 24 older and 25 younger adults. In all analyses, *p* ≤ 0.01 unless otherwise reported. We first examined the familiarity rating data. Overall, familiarity ratings were high (*M* = 4.5, SEM = 0.065), suggesting all participants were familiar with the idioms. Older adults (*M* = 4.76, SEM = 0.09) reported being more familiar with the idioms than younger adults (*M* = 4.26, SEM = 0.09), *F*_(1, 47)_ = 14.86, η^2^_p_ = 0.24.

Turning to the recognition data, an omnibus 3 × 2 × 2 ANOVA was performed with item type (studied, critical foil, filler) and phrase type (II, LE) as within subjects factors and age (younger/older) as a between subjects factor. The main effect of item type was significant, *F*_(2, 94)_ = 439.43, η^2^_p_ = 0.90. All pairwise comparisons were significant. IIs were endorsed as old more than LEs, *F*_(1, 47)_ = 24.59, η^2^_p_ = 0.34. The item type by phrase type interaction was significant, *F*_(2, 94)_ = 9.38, η^2^_p_ = 0.17, as was the three-way interaction, *F*_(2, 94)_ = 4.57, *p* = 0.013, η^2^_p_ = 0.09. No other effects were significant, all *F*s < 1.0. For the sake of brevity, we focus on the higher order interaction and report follow-up analyses for hits and false alarms.

Hit rates were submitted to a 2 (II-II vs. LE-LE) × 2 (age) mixed ANOVA. The interaction was significant, *F*_(1, 47)_ = 4.27, *p* = 0.044, η^2^_p_ = 0.08. Younger adults had slightly higher hit rates to IIs (*M* = 0.77, SEM = 0.04) than to LEs (*M* = 0.69, SEM = 0.04), *t*_(24)_ = 1.94, *p* = 0.064, whereas older adults had numerically higher hit rates to LEs (*M* = 0.76, SEM = 0.04) than intact items (*M* = 0.71, SEM = 0.04), *p* = 0.31. No other effects were reliable, both *F*s < 1.

False alarms were submitted to a 2 (condition at test: intact vs. LE) × 2 (foil type: critical foil, filler) × 2 (age) mixed ANOVA (see Table 3). The effect of foil type was significant, *F*_(1, 47)_ = 46.41, η^2^_p_ = 0.50, reflecting higher false alarm rates to critical foils (*M* = 0.20, SEM = 0.02) than to fillers (*M* = 0.10, SEM = 0.01). The effect of condition was significant, *F*_(1, 47)_ = 37.98, η^2^_p_ = 0.45, with IIs (*M* = 0.22, SEM = 0.02) judged as old more than LEs (*M* = 0.08, SEM = 0.01). Importantly, these effects were qualified by a condition by foil type interaction, *F*_(1,47)_ = 7.04, η^2^_p_ = 0.13. The difference between LE-II foils and intact fillers (0.17) was almost twice as large as the difference between II-LE foils and LE fillers (0.09). Thus, studying literal equivalents increased the accessibility or familiarity of the idioms with which they shared the literal meaning, whereas IIs did not drive up false alarm rates to their respective LEs as much. No other effects were significant, all *F*s < 1.7, *p*s > 0.20.

**Table 3:** **Average proportion of “old” responses as a function of age and item type (Standard deviations in parentheses)**.

	**Hit rates**		**False alarm rates**			
	**II-II**	**LE-LE**	**LE-II**	**II-LE**	**Foil II**	**Foil LE**
Young adults	0.71 (0.22)	0.69 (0.21)	0.27 (0.17)	0.12 (0.09)	0.10 (0.08)	0.05 (0.08)
Old adults	0.76 (0.23)	0.77 (0.17)	0.31 (0.19)	0.12 (0.13)	0.18 (0.19)	0.05 (0.07)
Average	0.74 (0.20)	0.72 (0.22)	0.29 (0.18)	0.12 (0.11)	0.14 (0.15)	0.05 (0.07)

These results suggest that the figurative meaning of familiar idioms is activated by phrases that share the literal meaning. The activation in semantic memory presumably occurred during the encoding phase, when spreading activation from lexical units in the LE increased the idiom's accessibility and resulted in the storage of the figurative meaning. However, when the idiom was studied, the corresponding LE was less likely to receive as substantial a boost in activation or accessibility, suggesting that idioms are more likely to be processed figuratively than literally. LEs, however, are processed via their constituent words and these, due to spreading activation processes, activate related neighbors, including the idiom itself. Thus, the presence of episodic and semantic traces jointly accessed at encoding for LE-IIs might contribute to heightened errors.

Idioms, however, presumably are processed figuratively *and* literally (e.g., Cacciari and Tabossi, 1988). If idioms were processed solely figuratively, errors to literal equivalents should be relatively similar for both critical foils and fillers, in that neither type of item would have received a boost in accessibility (i.e., *kick the bucket* would not affect *kick the pail*). However, if the idioms were also stored in their literal form, false alarms to LEs when the related idiom had been studied should be greater than false alarms to filler LEs (i.e., *kick the bucket* should result in some activation of *kick the pail*). Consistent with the idea that both literal and figurative meanings were stored during encoding, significantly more errors were made to II-LE items (*M* = 0.12, SEM = 0.02) than to LE fillers (*M* = 0.05, SEM = 0.01), *F*_(1, 47)_ = 13.80, η^2^_p_ = 0.23, and this effect did not vary with participant age, *F* < 1.

## Discussion

The goal of the present study was to explore how idioms are processed by examining how they are stored in memory; specifically, whether both literal and figurative meanings are accessed and retained in a comparable fashion, and the extent to which semantically similar LEs (e.g., *kick the pail*) increase the accessibility and/or availability of familiar idioms (e.g., *kick the bucket*). Younger and older participants studied lists of idioms and LEs and then completed a recognition test. Overall, hit rates were high and false alarms to fillers were low, suggesting participants were able to discriminate between studied and new items. Consistent with our hypothesis, relative to filler idioms and LEs, false alarms to the critical foils were higher, suggesting that prior study of related items did increase the accessibility or activation of the foils (cf. Roediger and McDermott, 1995). Critically, however, false alarms in the LE-II condition were significantly higher than those in the II-LE condition, suggesting that LEs provide access to the idiom more than the converse. Errors in the LE-II condition may reflect the activation of the figurative meaning during encoding: studying *kick the pail* increased the activation or accessibility of *kick the bucket*. Furthermore, II-LE errors exceeded errors to filler LEs, suggesting that studying *kick the bucket* did increase memory errors to *kick the pail*, presumably because of the shared literal meaning. This finding converges with previous evidence suggesting that idioms are processed and stored figuratively *and* literally (e.g., Cutting and Bock, 1997).

These results are also consistent with theories that propose that both literal and figurative meanings are activated (e.g., Cacciari and Tabossi, 1988), at least when there is no biasing context (cf. Rommers et al., 2013). This activation persists for at least several minutes, directly influencing the accessibility of phrases—both idioms and Les—that share literal meanings. The fact that both critical conditions (LE-II and II-LE) resulted in increased errors relative to the fillers suggests that the individual words, even in familiar idioms, are stored and processed for their literal meaning. As noted in the Introduction, equivalent rates of false alarms to LE-II and II-LE foils would have supported the idea that all phrases are stored literally and the basic gist was preserved; thus, foils that preserved the meaning of the idiomatic phrase (for example, the meaning of “kicking a pail or bucket”) would be equally likely to be falsely recognized (cf. Sachs, 1967). However, errors in the two conditions were not equivalent; errors in the LE-II condition were significantly higher than those in the II-LE condition, suggesting that accessing the figurative meaning of idiomatic expressions occurs in an obligatory fashion, not only when the idiom itself is studied, but also when a LE is processed. This is consistent with a spreading activation account, whereby activation at the lexical level of individual units or words activates related neighbors including the component words of the idiom itself. In other words, studying *kick the pail* activated *kick the bucket*, and the latter appears to have become part of the episodic trace. Thus, lexical access in semantic networks, as a result of spreading activation, resulted in the formation of episodic memory traces, which in turn resulted in memory errors on a recognition test. Such a finding is consistent with hybrid views of idiom processing, according to which both literal and figurative processing occurs.

Literal equivalents increased the accessibility of idiomatic expression and vice versa. However, simple spreading activation at the lexical level cannot solely explain the critical difference between LE-II and II-LE foils, because the activation was presumably bi-directional between LEs and IIs. If lexical level activation were the only factor, as noted above, equivalent errors would have been observed in both conditions. What we are suggesting is that the activation in semantic networks resulted in the indirect activation of the figurative meaning, and this was stored, with the literal meaning, and resulted in the inflated false alarms.

However, it is important to note that associative strength is a critical factor in determining the spread of activation in semantic networks (e.g., Roediger et al., 2001b). The critical constraint in selecting the nouns for LEs was to select items that preserved, as much as possible, the meaning of the literal interpretation of the idiom. Thus, we primarily selected synonyms, where possible, or items that shared features (e.g., *grasp-grip* for the idiom *get a grip* or *bull-cow* for the idiom *have a cow*). Pairwise cosine values from the Latent Semantic Analysis database (LSA; Landauer and Dumais, 1997) were calculated to assess the similarity. Briefly, LSA cosines are a measure of semantic similarity obtained by comparing the usage and frequency of pairs of items across a large database. Higher cosine values reflect more similarity, where similarity captures factors such as occurrence in similar contexts. The average LSA cosine for the items used in the present study was 0.41, which indicates a fairly high level of similarity.

To ensure that the difference in false alarm rates was not due to differences in associative strength between LE-II items and II-LE items, bidirectional (from the LE to the idiom and vice versa) associative strength values between the nouns were obtained from the University of South Florida Free Association Norms (Nelson et al., 1998). Values were available for 54 of the 60 pairs of items. The mean associative strength from the LE noun to the idiom noun was significantly higher (*M* = 0.16, *SD* = 0.24) than the opposite direction (*M* = 0.09, *SD* = 0.16), *t*_(53)_ = 2.10, *p* = 0.04. In additional analyses, the five pairs with the largest difference in associative strength were removed. This resulted in a set of items with matched associative strength: an average of 0.11 (*SD* = 0.17) for the LE to idiom direction and 0.10 (*SD* = 0.17) for the idiom to LE direction, *t* < 1. Using only this subset of items, the ANOVA comparing critical foil and non-studied foil false alarms was conducted. Importantly, the 2-way interaction between condition and foil type remained significant, *F*_(1, 47)_ = 5.03, *p* = 0.03, η^2^_p_ = 0.10. This confirms that the higher rate of false alarms to idioms from studying LEs relative to the false alarms to LEs after studying idioms was not driven by a handful of items that were strongly associated. For hit rates, analyses on the matched set of items revealed a marginally significant interaction, *F*_(1, 47)_ = 3.84, *p* = 0.056, η^2^_p_ = 0.076, similar to the one observed with the full set of stimuli. Thus, the difference between LE-II and II-LE items was not simply due to differences in activation of related items.

As noted in the Introduction, reliable memory errors can be elicited by using a list-learning paradigm. In some studies using the DRM paradigm, false recognition rates are as high as hit rates (e.g., Roediger and McDermott, 1995). The false alarm rates observed in the present study were noticeably lower than those obtained in DRM studies. Because only one “related” item was studied (i.e., the paired LE or II, depending on the critical foil condition) compared to the 12–15 items typically used in DRM studies, these errors provide further evidence in support of the powerful role of semantic activation in episodic memory tasks and demonstrate reliable false memory following study of a single related item.

An alternative interpretation of the present results is that the increased false alarms in the LE-II condition relative to the II-LE condition simply reflected differences in familiarity—in other words, the effect we observed might be similar to a word frequency mirror effect (e.g., Balota and Neely, 1980; Glanzer and Adams, 1985), whereby higher frequency items are more likely to be incorrectly recognized due to higher baseline familiarity levels, whereas lower frequency items are more correctly recognized, due to their distinctiveness (Joordens and Hockley, 2000; Reder et al., 2000; Coane et al., 2011). The idioms were more likely to be familiar compared to the LEs (Popiel and McRae, 1988), suggesting this might be the case. Two lines of evidence argue against this explanation. First, hit rates overall did not differ between LEs and idioms; thus, there was no full-blown mirror effect. Although younger adults had slightly higher hit rates to intact idioms than to LEs, older adults, who reported having greater familiarity with the idioms, showed the opposite effect. Moreover, neither within-group comparison was significant. Thus, familiarity alone was not driving hit rates. Second, although false alarms to fillers and to critical foils showed the same pattern (i.e., more false alarms to idioms than LEs), the presence of the interaction suggests that the stored figurative meaning, accessed during the study phase, contributed to errors above and beyond the familiarity effects. However, studying an LE did indeed influence the accessibility of the idiom itself, as reflected by the almost two-fold increase in errors in the LE-II condition relative to the II-LE condition compared to the difference in errors between filler LEs and idioms.

Nonetheless, because of the large effects of word frequency in episodic recognition, we did examine the objective frequency of the nouns used in LEs and IIs using the Hyperspace Analog to Language database (Lund and Burgess, 1996) accessed through the English Lexicon Project database (Balota et al., 2007). On average, nouns in the idiomatic expressions had higher estimated frequency (*M* = 9.91, *SD* = 1.48) compared to the nouns used in the LEs (*M* = 8.92, *SD* = 1.64), *t*_(59)_ = 4.29, *p* < 0.001. To ensure that the interaction between foil type and condition was not driven by baseline differences in word frequency, 13 pairs of items were omitted from analyses to balance the LEs and idioms in terms of noun frequency (*M* = 9.31, *SD* = 1.45, and *M* = 9.66, *SD* = 1.43, respectively), *t*_(46)_ = 1.68, *p* = 0.10. The ANOVA comparing critical foils and fillers was performed after removing these items. The interaction between foil type and condition was marginally significant, *F*_(1, 47)_ = 3.52, *p* = 0.067, η^2^_p_ = 0.07. Although not significant, possibly due to the reduction in power, it is important to note that the difference in false alarm rates between LE-II foils and intact fillers (*M* = 0.15) was still about twice the magnitude as the difference between false alarm rates to II-LE foils and LE fillers (*M* = 0.076). Thus, even after removing a subset of items in which the idiom noun was higher in frequency than the LE noun, the robust increase in errors in the LE-II condition still remained relative to the II-LE condition, suggesting that frequency of the component nouns was not solely driving the effect.

The results might have, in part, reflected a trade-off between familiarity and distinctiveness. Distinctiveness refers to the extent to which an item is *different* relative to other to-be-remembered items (Hunt and Elliott, 1980) and generally results in enhanced memory performance. In this context, in which LEs were mixed with idioms, the LEs might have appeared distinctive because they were similar in structure and literal meaning to the idioms, but were clearly not the intact idiomatic expressions. The LEs in the present study therefore may have been more distinctive than the very familiar idioms, and this might have enhanced hit rates and reduced false alarm rates to these items. Although we cannot rule this out, the differential rates of false alarms to fillers and critical items does suggest that studying phrases with shared literal meanings does affect errors to related items.

Interestingly, the effects of participant age were small. Older adults reported being more familiar with the idioms, despite the fact that norming was conducted with college-aged participants. In the recognition data, there were no consistent age effects. Because of age-related declines in episodic memory coupled with relatively preserved semantic memory, as well as age-related deficits in controlled and inhibitory processes, one might have expected older adults to show larger effects of the foil type manipulation (i.e., more errors in the LE-II condition due to the spread of activation from LEs to idioms and deficits in source monitoring). Particularly in light of the strong familiarity older adults reported for the idioms used here, the relative similarity in performance across age groups is intriguing. There is evidence that older adults' memory deficits can be reduced when the to-be-learned material is more meaningful or relevant (Charles et al., 2003; Gutchess et al., 2007), thus it is possible that the use of familiar idioms might have been more engaging for this group than unrelated word lists. Supporting this interpretation, older adults had slightly higher hit rates for LEs than idioms, suggesting that these items may have captured their attention and promoted deeper processing. For example, older adults might have seen *kick the pail* and consciously processed the fact that this is an incorrect variant of the idiom, thus resulting in a stronger memory trace. Their familiarity with the idioms might have then encouraged them to consciously retrieve the “correct” version of the phrase (i.e., *kick the bucket*), thereby increasing item-specific processing for the LEs. However, if this were the case we might have expected older adults to have higher false alarms to the LE-II items relative to younger adults due to source monitoring errors that are more prominent in aging (Hashtroudi et al., 1989; Dywan and Jacoby, 1990). It therefore remains unclear whether the equivalent performance in younger and older adults is due to the fact that the specific stimuli used here promote additional richness of encoding/additional environmental support (Craik, 1986), or whether knowledge about idioms is in some way special and more resistant to age-related declines than other types of material, though there is evidence to support the later interpretation. For example, older adults can recognize metaphors, another form of figurative language, as well as, or better than young adults (e.g., Bonnaud et al., 2002), and a recent study failed to find evidence of age-related declines in a number of tasks assessing idiom comprehension (Hung and Nippold, 2014). Such findings suggest that the additional linguistic experience accumulated by older adults might support processing of language in general, and figurative language in particular. Future work, however, is necessary to disentangle to what extent idiom processing changes over the lifespan and in what ways.

The present study should be interpreted in light of several limitations. First, we did not systematically manipulate idiom decomposability or transparency (the extent to which the figurative meaning can be derived from the constituent words). The stimuli used included both decomposable idioms (e.g., *hold your horses*, *miss the boat*) and non-decomposable idioms (e.g., *bite the dust*, *break the ice*) according to the norms of Titone and Connine (1994b). However, decomposability ratings were not available for more than half of the items used, thus we were not able to examine whether this factor, as well as other factors like predictability (the extent to which the final word in an idiom is predictable given the earlier words), might affect the results. Future work should manipulate these factors to assess whether there are also differences in how decomposable and non-decomposable idioms are remembered.

Second, the present study focused rather narrowly on processing of a specific form of idiomatic expressions (i.e., verb-noun phrases). However, the results might be of interest to researchers in other areas of figurative language and pragmatics. Recent work in this area suggests that, even in figurative language such as metaphors and irony, there might be ongoing processing at the literal level (see Carston, 2010). Briefly, theories such as Relevance Theory propose that meaning of individual lexical entries cannot exist out of context and that meaning emerges in each given communication context where the word appears. In the present context, this would imply that the meaning of the word *bucket* is not “purely” literal or “purely” figurative; rather, the word (as in a lexical entry in a mental lexicon) might be best thought of as a “grab bag” of meaning, with the appropriate meaning, as determined by the broader linguistic context, being preferentially accessed and activated (Carston, 2012). In other words, it might not make sense to consider literal and figurative meanings as opposite or mutually exclusive, but as elements that contribute to how language is understood in context. The present results, showing that related noun phrases activate one another, even when one of them is a “fixed” expression such as an idiom, confirm that meaning is not fixed, but flexible and dynamic.

## Conclusion

The paradigm used here differs somewhat from the online measures used in many studies that carefully track the time course of idiom processing to assess at what point in processing literal and figurative meanings are accessed. Here, the use of a delayed memory test allowed us to use errors as a means of examining underlying processes. As argued by Roediger (1996) and Schacter (1999), careful analysis of memory errors can help one understand the underlying organization of the systems that support memory performance. This logic is evident in research on perceptual illusions (e.g., Rock, 1984) and can be fruitfully applied to other domains. By examining errors in a memory test, the present study provides novel evidence for the activation and storage of both literal and figurative meanings of idioms in two age groups. The results were consistent with models of idiom processing that allow for both figurative and literal processing to occur.

### Conflict of interest statement

The authors declare that the research was conducted in the absence of any commercial or financial relationships that could be construed as a potential conflict of interest.
