# Supplementary material for: False memory for idiomatic expressions in younger and older adults: evidence for indirect activation of figurative meanings
Source: Front Psychol. 2014 Jul 21;5:764. doi: 10.3389/fpsyg.2014.00764 (PMC4104352; doi:10.3389/fpsyg.2014.00764)
Supplement: Supplementary file 1 [file DataSheet1.DOCX]

**Appendix**

All the idioms, their literal equivalents, the directional associative strength (AS) between them, and the familiarity ratings given to idioms during the experimental phase (on a scale of 1 = not at all familiar to 5 = very familiar)

| **Idiom** | **Literal Equivalent** | **AS from LE to II** | **AS from II to LE** | **Familiarity (Young Adults)** | **Familiarity (Old Adults)** | **Average Familiarity** |
| --- | --- | --- | --- | --- | --- | --- |
|  |  |  |  |  |  |  |
| bite the dust | bite the dirt | .00 | .00 | 4.67 | 4.76 | 4.71 |
| bite your tongue | bite your lip | .00 | .00 | 4.67 | 4.96 | 4.82 |
| blow your top | blow your cap | .00 | .00 | 3.46 | 4.76 | 4.12 |
| break all ties | break all links | .00 | .00 | 5 | 4.92 | 4.96 |
| break a leg | break an arm | .67 | .50 | 3.58 | 4.16 | 3.88 |
| break the ice | break the snow | .00 | .00 | 4.96 | 4.64 | 4.8 |
| burn your bridges | burn your walkways | .00 | .00 | 4.08 | 4.76 | 4.43 |
| bury the hatchet | bury the ax | .03 | .47 | 3.13 | 4.92 | 4.04 |
| clear the air | clear the cloud | .00 | .00 | 4.42 | 4.88 | 4.65 |
| cross your fingers | cross your toes | .00 | .09 | 4.96 | 4.92 | 4.94 |
| cut the cheese | cut the butter | .00 | .00 | 4.46 | 3.88 | 4.16 |
| cut your losses | cut your defeats | .00 | .00 | 3.83 | 4.76 | 4.31 |
| drag one's feet | drag one’s legs | .00 | .00 | 3.58 | 4.68 | 4.14 |
| draw a blank | draw a gap | NA | .00 | 4.83 | 4.88 | 4.86 |
| drop the ball | drop the balloon | .00 | .00 | 4.13 | 4.68 | 4.41 |
| eat your words | eat your verbs | .00 | .00 | 4.29 | 4.76 | 4.53 |
| face the music | face the song | .21 | .07 | 3.54 | 4.88 | 4.22 |
| fly the coop | fly the pen | .00 | NA | 3.08 | 4.84 | 3.98 |
| foot the bill | foot the check | .02 | .03 | 3.42 | 4.64 | 4.04 |
| get a grip | get a grasp | .03 | .02 | 4.88 | 4.76 | 4.82 |
| get cold feet | get cold toes | .53 | .47 | 4.71 | 4.88 | 4.8 |
| get the picture | get the photograph | .58 | .06 | 4.83 | 4.92 | 4.88 |
| have a cow | have a bull | .56 | .10 | 4.29 | 4.44 | 4.37 |
| have a crush | have a crash | .00 | .00 | 4.54 | 4.64 | 4.59 |
| hit the sack | hit the bag | .11 | .44 | 4.71 | 4.84 | 4.78 |
| hit the roof | hit the ceiling | .35 | .17 | 2.79 | 4.72 | 3.78 |
| hit the books | hit the pages | .45 | .04 | 4.38 | 4.8 | 4.59 |
| hold your horses | hold your ponies | .75 | .11 | 4.92 | 4.88 | 4.9 |
| join the club | join the group | .00 | .00 | 4.67 | 4.92 | 4.8 |
| jump the gun | jump the pistol | .77 | .06 | 4.29 | 4.88 | 4.59 |
| kick the bucket | kick the pail | .50 | .22 | 4.21 | 4.92 | 4.57 |
| learn the ropes | learn the strings | .23 | .13 | 4.58 | 4.88 | 4.73 |
| lend a hand | lend a finger | .27 | .36 | 4.88 | 4.88 | 4.88 |
| lend an ear | lend an eye | .06 | .01 | 4.25 | 4.88 | 4.57 |
| make a killing | make a slaying NA | | .00 | 3.88 | 4.84 | 4.37 |
| make the cut | make the chop | .00 | .00 | 4.04 | 4.68 | 4.37 |
| miss the boat | miss the canoe | .43 | .02 | 3.71 | 4.76 | 4.24 |
| pick a bone | pick a muscle | .01 | .01 | 3.71 | 4.4 | 4.06 |
| play the game | play the toy | .09 | .00 | 3.88 | 4.96 | 4.43 |
| pop the question | pop the query NA | | .00 | 4.54 | 4.84 | 4.69 |
| pull the plug | pull the stopper | .00 | .00 | 4.13 | 4.92 | 4.53 |
| push the envelope | push the letter | .07 | .49 | 3.71 | 4.56 | 4.14 |
| rack your brain | rack your mind | .37 | .04 | 4.5 | 4.88 | 4.69 |
| rock the boat | rock the canoe | .43 | .02 | 4.5 | 4.88 | 4.69 |
| see the light | see the lamp | .77 | .02 | 4.29 | 4.88 | 4.59 |
| set the pace | set the gait | NA | NA | 4.46 | 4.76 | 4.61 |
| set the stage | set the platform | .18 | .03 | 4.42 | 4.64 | 4.53 |
| settle a score | settle a grade | .02 | .07 | 4.13 | 4.68 | 4.41 |
| shoot the breeze | shoot the wind | .12 | .61 | 3.29 | 4.88 | 4.1 |
| smell a rat | smell a mouse | .09 | .32 | 3.83 | 4.84 | 4.35 |
| spill the beans | spill the peas | .08 | .03 | 4.92 | 4.96 | 4.94 |
| steal the show | steal the play | .00 | .00 | 4.58 | 4.84 | 4.71 |
| tackle a problem | tackle a difficulty | .02 | .02 | 4.29 | 4.72 | 4.51 |
| take a beating | take a bashing | NA | NA | 4.17 | 4.56 | 4.37 |
| test the water | test the milk | .00 | .00 | 4.42 | 4.84 | 4.63 |
| tie the knot | tie the bow | .00 | .00 | 4.5 | 4.92 | 4.71 |
| turn the tables | turn the desks | .09 | .02 | 4.13 | 4.84 | 4.49 |
| walk the line | walk the row | .00 | .00 | 3.67 | 4.44 | 4.06 |
| wear the pants | wear the trousers | .00 | .00 | 4.42 | 4.76 | 4.59 |
| weigh your options | weigh your possibilities | .00 | .00 | 4.71 | 4.84 | 4.78 |
